# Can Improved Farm Biosecurity Reduce the Need for Antimicrobials in Food Animals? A Scoping Review

**DOI:** 10.3390/antibiotics12050893

**Published:** 2023-05-11

**Authors:** Pankaj Dhaka, Ilias Chantziaras, Deepthi Vijay, Jasbir Singh Bedi, Iryna Makovska, Evelien Biebaut, Jeroen Dewulf

**Affiliations:** 1 Faculty of Veterinary Medicine, Department of Internal Medicine, Reproduction and Population Medicine, Ghent University, Salisburylaan 133, 9820 Merelbeke, Belgium; 2 Centre for One Health, College of Veterinary Science, Guru Angad Dev Veterinary and Animal Sciences University, Ludhiana 141004, India; 3 Department of Veterinary Public Health, College of Veterinary and Animal Sciences, Thrissur 680651, India

**Keywords:** antimicrobial usage, cattle, farm biosecurity, herd health, pigs, poultry

## Abstract

Limited and judicious antimicrobial usage (AMU) is considered the key to saving the success of human and veterinary medicine in treating infections. With the limited alternatives for antimicrobials, farm biosecurity (and herd management) is considered a promising tool to mitigate the non-judicious AMU and to maintain animal health, production, and welfare. The present scoping review aims to analyse the effect of farm biosecurity on AMU in livestock systems and formulate recommendations. Peer-reviewed manuscripts published between 2001–2022 were analyzed using the PRISMA framework using PubMed, Scopus, and Science Direct databases. After applying the inclusion criteria, 27 studies were found to assess the effect of farm biosecurity (or management practices) on AMU at the herd/farm level in quantitative/semi-quantitative terms. These studies were carried out in 16 countries, of which 74.1% (20/27) were from 11 European countries. The highest number of studies were from pig farms [51.8% (14/27)], followed by poultry (chicken) farms [25.9% (7/27)], cattle farms [11.1% (3/27)], and a single study from a turkey farm. Two studies include both pig and poultry farms. Most of the studies were cross-sectional [70.4% (19/27)], seven were longitudinal, and one was a case-control study. Complex interactions were observed among factors influencing AMU, such as biosecurity measures, farm characteristics, farmers’ attitudes, availability of animal health services, stewardship, etc. A positive association between farm biosecurity and reduction in AMU was observed in 51.8% (14/27) of the studies, and 18.5% (5/27) showed that improvement in farm management practices was associated with a reduction in AMU. Two studies highlighted that coaching and awareness among farmers might lead to a decrease in AMU. A single study on economic assessment concluded biosecurity practices as a cost-effective way to reduce AMU. On the other hand, five studies showed an uncertain or spurious association between farm biosecurity and AMU. We recommend the reinforcement of the concept of farm biosecurity, especially in lower- and middle-income countries (LMICs). Further, there is a need to strengthen the evidence on the association between farm biosecurity and AMU in region- and species-specific farm settings.

## 1. Introduction

Antibiotics are one of the most impactful public health-related discoveries of the 20th century [1,2]. Apart from its vital contribution to human health, the application of antimicrobials in the livestock sector played a significant role in upgrading animal health, production, and welfare [3]. Nevertheless, along with noteworthy benefits, indiscriminate antimicrobial usage (AMU) has been the driver for antimicrobial resistance (AMR) selection, which is continuously threatening the global public and animal health systems [4]. Previous studies have proposed a possible link between AMU in animal farming and the emergence of resistant pathogens that can affect the health of both animals and humans [5,6]. In China in 2013, Zhang et al. (2015) observed that after the metabolism of 36 antibiotics with a total usage of 92,700 tons, the total excretion amount was 54,000 tons (84% excreted by animals and 16% by humans), and eventually the emission to the environment was 53,800 tons (46% received by water and 54% to the soil) [7]. High AMU in animal husbandry can lead to resistant organisms and drug residues in animal-derived products [8,9]. Furthermore, using antimicrobials in animal products that are also employed in human medicine can lead to cross-resistance development [5,10]. This underscores the importance of reducing AMU in animal husbandry to alleviate selection pressure on bacteria and safeguard animal and human health [11].

Factors influencing AMU in animal farming are multifaceted and vary from one region to another [12,13]. Although measures have been taken to reduce the non-judicious use of antimicrobials through various policies and guidelines, using these drugs for non-therapeutic purposes, such as growth promotion, prophylaxis, and metaphylaxis, remains prevalent in many regions across the world [14]. The non-therapeutic use of antimicrobials is primarily intended to mask suboptimal farming conditions such as inferior feed, unclean water, improper housing, stressful transportation, poor farm hygienic conditions, and improper vaccination and deworming schedules [15]. Notably, variations in the amount, the timing of administration, and antimicrobial classes used can be observed both within and across livestock-rearing systems [16]. Furthermore, collecting farm-level AMU data is challenging, and results can vary depending on the collection methods and methodologies used [17].

Farm biosecurity is considered a valuable tool to limit the non-judicious use of antimicrobials and promote animal health, production, and welfare [9,18]. It involves measures to prevent the introduction and spread of infectious agents. It includes practices such as restricted movements, animal quarantine and isolation, fencing, transport, cleaning and disinfection protocols, and diagnostic facilities [19]. By implementing farm biosecurity and herd management practices, disease outbreaks and the incidence of infectious diseases among farm animals can be reduced, potentially mitigating the need for antimicrobial treatments and the risk of AMR development [20]. In addition, these measures are cost-effective for preventing infectious diseases in livestock [20,21], although there is limited research on their impact on AMU [22].

Adoption of farm biosecurity practices varies widely among geographic regions, social groups, and livestock production chains, with factors such as farmers’ socio-demographic characteristics and attitudes, farm’s physical and economic constraints, access to information, trust between farmers and animal health authorities, and the belief that biosecurity is primarily a government responsibility influencing farmers’ motivation to invest in biosecurity components [23,24,25]. Despite the practical advantages of farm biosecurity on animal health and welfare, farmers and animal health professionals in resource-limited regions may still be hesitant about its efficacy in substituting or replacing non-judicious AMU practices [18,26]. Therefore, it is crucial to assess herds’ biosecurity levels at regional and national levels and analyze the associations between biosecurity scores, management factors, and AMU to build trust among livestock producers, animal health professionals, and policymakers. This review aims to explore the relationship between antimicrobial usage (AMU) in livestock production and farm biosecurity measures by analyzing the existing literature. Through a thorough examination of available data, this review aims to provide a comprehensive understanding of the potential benefits of farm biosecurity in reducing AMU.

## 2. Methodology

The manuscripts published between 2001–2022 were analyzed for conducting a scoping review as per the procedures established by the Preferred Reporting Items for Systematic Reviews and Meta-Analyses (PRISMA) statement extension for scoping reviews (PRISMA-ScR) (checklist provided as Supplementary Table S1) [27]. The study’s research question was, “*Is there an association between farm biosecurity and antimicrobial usage (AMU) in food animals?*”. The articles were searched from three electronic databases (PubMed, Scopus, and Science Direct), all through “Title-Abstract-Keywords”. The inclusion criteria were (a) studies published in peer-reviewed journals in the English language; (b) studies published between 2001–2022; and (c) studies which are assessing the effect of farm biosecurity measures (or herd management practices) on antimicrobial usage (AMU) in livestock systems (including poultry). The search was conducted from 16 December 2022 to 2 January 2023, and the search algorithms consisted of various combinations of keywords to enforce the Boolean search strategy. The used keywords and their strings [i.e., (“biosecurity” OR “farm biosecurity” OR “animal biosecurity” OR “preventive veterinary medicine” OR “herd health management”) AND (“antimicrobial resistance” OR “antibiotic resistance” OR “antimicrobial usage” OR “antibiotic usage”) AND (“Livestock” OR “poultry” OR “farm” OR “animal production” OR “cattle” OR “dairy animals” OR “dairy cattle” OR “beef production” OR “beef cattle” OR “buffalo*” OR “pig” OR “swine” OR “goat” OR “sheep” OR “chicken” OR “broiler” OR “layer” OR “turkey” OR “duck”)] were adjusted as per the targeted electronic database (Supplementary Table S2).

Only peer-reviewed original research articles were included in the analysis to gather evidence related to the research question. Therefore, literature reviews, editorials, commentaries, and papers published in languages other than English were excluded. Articles that only explored farm biosecurity (or management issues) without mentioning the effect of AMU on livestock or that only mentioned AMU practices without mentioning the status of farm biosecurity (or management practices) were also excluded. Additionally, review articles were explored to identify any additional original research articles not identified through the search algorithm.

### 2.1. Data Extraction

An initial screening of the articles was independently carried out by two researchers using the inclusion criteria. Then, the selected articles were moved to Zotero reference management software for full-text analysis. Finally, all authors reviewed the selected manuscripts and revision was made based on the individual’s feedback. After selecting the relevant articles, data were extracted and recorded in a Microsoft Excel worksheet (Microsoft Corp., Santa Rosa, CA, USA). The data include the country where the study was conducted, year and duration of the study, animal species and farm size, study type, data on AMU, data on-farm biosecurity, the association between farm biosecurity and AMU (if any), and study citation.

### 2.2. Data Analysis

The data from the studies included in this analysis were examined based on various factors, including geographic location, study type, livestock species, and the relationship between farm biosecurity or management practices and AMU. A hypothetical causal diagram was created to understand better the impact of farm biosecurity or management practices on AMU at the farm/herd level, which outlined direct and indirect pathways involving one or more intervening variables. This analysis formulated recommendations to further reduce non-judicious AMU in livestock systems based on carefully evaluating the included studies. 

## 3. Results and Discussion

### 3.1. Studies Characteristics

**Figure 1:**
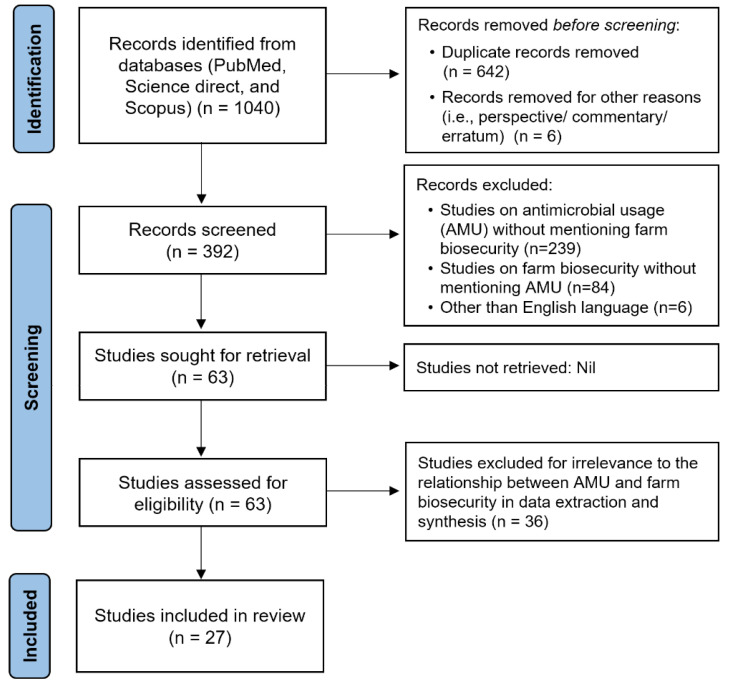
Flow chart of the systematic literature search on-farm biosecurity and its effect on antimicrobial usage.

The initial search hits resulted in extracting 1040 articles from PubMed, Science Direct, and Scopus. After the removal of duplicates and applying the inclusion criteria, a total of 63 articles were selected for full-text review. Out of 63 articles, 27 studies observed the effect of farm biosecurity (or management practices) on AMU at the herd/farm level in quantitative/semi-quantitative terms (Figure 1 and Table 1).

**Figure 2:**
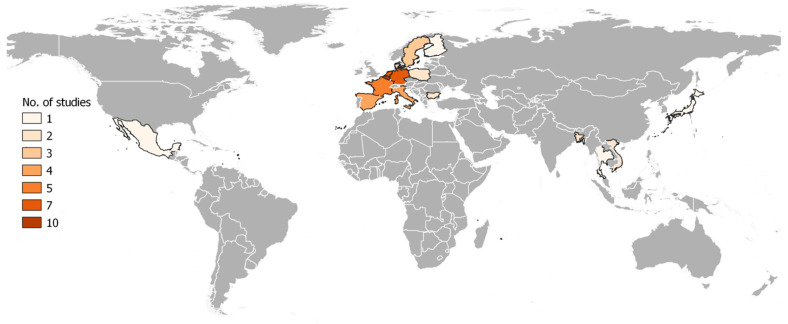
Geographic distribution of research on the correlation between farm biosecurity measures and antimicrobial usage in livestock production worldwide. Note: If a publication includes multiple countries, it has been counted once for each country included.

The selected studies (n = 27) were carried out among 16 countries across the globe (Figure 2), and most of these studies [74.1% (20/27)] were conducted in European countries (n = 11) (Table 1). In terms of targeted livestock species, the highest number of studies were carried out at pig farms [51.8% (14/27)], followed by poultry (chicken) farms [25.9% (7/27)], cattle farms [11.1% (3/27)], and a single study from a turkey farm. In addition, two studies include both pig and poultry farms. Among the 27 studies, the majority (70.4%, n = 19) were cross-sectional studies or observational surveys. In comparison, seven studies were longitudinal (including four with intervention measures), and one was a case-control study (Table 1).

**Table 1:** Characteristics of studies included for the analysis on the farm biosecurity and antimicrobial usage (AMU).

Sl.	Country/Groups	Livestock Type	Study Type	Number of Farms Involved	Study Year	Association of Farm Biosecurity Parameters and AMU	Reference
1.	Bangladesh *	Poultry (chicken) farms	Cross-sectional	768	2021	‘Chicken morbidity’ and ‘farm location’ were significantly associated with increased AMU.	[28]
2.	Bangladesh	Poultry (chicken) farms (layer and broiler)	Cross-sectional	140	2019	‘Separation of sick from healthy birds’ was significantly associated with reducing AMU. ‘Farms that used shallow water’ were more likely to use antimicrobials.	[26]
3.	Belgium	Pig farms	Longitudinal with intervention	Raising pigs without antibiotics (RWA) = 16; non-RWA = 12	2018–2021	After farm-specific coaching, for non-RWA farms, there was a reduction in AMU of 61%, 38%, and 23%, for the suckling piglets, fattening pigs, and sows, respectively.	[29]
4.	Belgium	Pig farms	Longitudinal with intervention	50	2010–2012	Costs incurred by new biosecurity measures (median +€3.96/sow/year), and new vaccinations (median €0.0/sow/year) didn’t exceed the cost reduction achieved by lowering AMU (median −€7.68/sow/year).	[30]
5.	Belgium	Pig farms	Longitudinal with intervention	61	2010–2014	Biosecurity improvement led to a 52% decrease in AMU for pigs from birth to slaughter and a 32% decrease for breeding animals based on treatment incidences (TIs).	[31]
6.	Belgium	Poultry (broiler) farm	Longitudinal with intervention	15	2012–2013	Farmers were advised to improve biosecurity after the first audit, and a second audit was conducted within a year to assess changes. Based on the second audit, the average AMU decreased by 29% (from 192 to 136 TIs).	[32]
7.	Belgium	Pig farms	Cross-sectional	95	2009–2011	Improved biosecurity scores were associated with lower AMU, as indicated by lower TIs. The ‘disease management’ and ‘farrowing/suckling’ factors were also negatively associated with TIs.	[33]
8.	European countries (Belgium and The Netherlands)	Poultry (broiler) and pig farms	Cross-sectional	30 poultry (broiler) and 30 pig farms	2017–2018	Dutch farms had better overall biosecurity than Belgian farms. However, Belgian farms had higher AMU in pig weaners, finishers, and broilers compared to Dutch farms.	[22]
9.	European countries (Germany, France, and Spain)	Turkey farms	Cross-sectional	60	2014–2016	The study showed unclear links between biosecurity and AMR, but some antimicrobial classes were positively associated with AMU and AMR.	[34]
10.	European countries (Belgium, France, Germany and Sweden)	Pig farms	Cross-sectional	227	2012–2013	Better external biosecurity was associated with lower AMU, while ‘shorter farrowing rhythm’ and ‘younger weaning age’ were linked to higher AMU.	[18]
11.	European countries (Belgium, France, Germany, and Sweden)	Pig farms	Cross-sectional	227	2012–2014	Compared to ‘regular farms’, the ‘top farms’ with high productivity and low AMU had a higher biosecurity status and fewer gastrointestinal symptoms in sucklers and clinical respiratory symptoms in fatteners.	[35]
12.	European countries (Belgium, Bulgaria, Denmark, France, Germany, Italy, The Netherlands, Poland and Spain)	Poultry (chicken) farms (broiler)	Cross-sectional	181	2014–2016	Antimicrobial-resistant genes (ARGs) were positively associated with corresponding AMU. Higher internal biosecurity correlated with more oxazolidinone ARGs, despite not being used in broiler production. Evidence was insufficient to support the hypothesis that biosecurity affects the spread of ARGs.	[36]
13.	European countries (Belgium, Bulgaria, Denmark, France, Germany, Italy, The Netherlands, Poland, and Spain)	Poultry (chicken) farms (broiler)	Cross-sectional	181	2014	Farm size, intensity, and having a hygiene lock were associated with lower AMU while obliging visitors to check in, vaccination protocols, and flock depopulation in steps were associated with higher AMU.	[9]
14.	Finland	Pig farms	Longitudinal	406	2011–2013	Poor farm management (unhygienic drinking equipment, poor pen condition, poor air quality, poor cleanliness) leads to increased antimicrobial TIs. However, this poor management can also lead to musculoskeletal disorders, tail biting, joint infections, and respiratory diseases associated with high AMU.	[37]
15.	Germany	Pig farms	Longitudinal	200	2011, 2013, 2014	Farm size, veterinarian, and farm category impacted TIs, while increased animal movement and pooling of animals from different stables increased the risk of infections and, thus, AMU.	[38]
16.	Germany	Pig farms	Cross-sectional	60	2012–2014	Lower external biosecurity scores are associated with more antimicrobial treatments in pigs, particularly for lameness and gastrointestinal diseases in suckling pigs and lameness, respiratory diseases, and skin symptoms in weaned and fattening pigs.	[39]
17.	Italy	Dairy cattle	Cross-sectional	34	2018–2020	Farms using replaceable bedding materials had lower AMU. No significant correlation between AMU and biosecurity/animal welfare scores was observed. The authors concluded that management factors have minimal effect on AMU.	[40]
18.	Italy *	Pig farms	Cross-sectional	14	2016	Antibiotics were frequently used to treat gastroenteritis in 57% of farms and prophylactic/metaphylactic use in 50%. Vaccination and good management practices were recommended for high- and medium-risk farms.	[41]
19.	Japan	Pig farms	Cross-sectional	38	2015	Higher external biosecurity scores and low-density isolated farms were linked to lower oral AMU. In addition, low post-weaning mortality, controlled pig flows, an all-in-all-out system, and good internal biosecurity were associated with lower AMU.	[42]
20.	Mexico	Poultry (chicken) farms (layers and broilers)	Cross-sectional	43	2017–2018	Stronger biosecurity measures were linked to lower AMU in a cluster of farms.	[43]
21.	The Netherlands	Dairy cattle	Longitudinal	94	2005–2012	Combining veterinarian awareness-raising on herd management and restrictive measures led to a reduction in AMU (−17% in 2012 vs. 2009), resulting in lower veterinary costs per cow.	[44]
22.	The Netherlands	Dairy cattle	Case-control	200 (100 high AMU and 100 low AMU)	2012–2013	Housing calves on partially slatted floors, high prevalence of respiratory disease, unfavorable Salmonella status, and lack of agreement with specific young stock management were associated with high AMU.	[45]
23.	Spain	Pig farms	Cross-sectional	37	2017–2018	High AMU may lead to increased biosecurity, while poor biosecurity may increase the need for antimicrobials. Organic-extensive farms have lower AMU due to less animal density and confinement.	[46]
24.	Sweden	Pig farms	Cross-sectional	60	2013	Low internal biosecurity is linked to high TI in weaners, fatteners, and adults, and external biosecurity linked to TI in weaners.	[47]
25.	Thailand *	Pig farms	Cross-sectional	114	2018	Small pig farms resisted colistin and fluoroquinolones, while medium-sized farms showed resistance to streptomycin.	[5]
26.	Vietnam *	Chicken (layer and broiler) and pig farmers	Cross-sectional	540 each	2018	The lack of veterinary services, access to over-the-counter antimicrobials, and insufficient farm biosecurity were linked to high AMU. In addition, educating farmers was found to impact AMU compliance.	[13]
27.	Vietnam *	Poultry (chicken) farms (layer/broiler/roosters)	Cross-sectional	125	2020	The farms under the company’s contract (Group A) had good biosecurity measures, infrastructure, and access to veterinarians during health emergencies, reducing AMU in the study region.	[48]

* No quantitative farm biosecurity scoring was carried out in these studies; however, authors associated the farm management (or biosecurity) parameters with AMU or discussed them in context with AMU.

### 3.2. Farm Biosecurity (or Management) Factors Affecting Antimicrobial Usage (AMU)

The present scoping review found a limited number of studies (n = 27) that have investigated the association between farm biosecurity and AMU at the farm or herd level. Out of these, 51.8% (14/27) showed a positive association between the implementation of farm biosecurity and reduction in AMU, while 18.5% (5/27) demonstrated that improved farm management practices were associated with lower AMU. Furthermore, two studies indicated that coaching and awareness among farmers could lead to reduced AMU, while a single economic assessment study concluded that implementing biosecurity practices is a cost-effective strategy for reducing AMU (Table 1). On the other hand, five studies reported an uncertain or spurious association between farm biosecurity and AMU, which could be attributed to confounders such as recent outbreaks, underreporting of AMU, misclassification, or missing information on AMU and/or biosecurity [9,34,36,40,46] (Table 1).

The review summarizes the current scientific evidence on the relationship between biosecurity measures and AMU reduction in livestock production. Biosecurity measures such as following an all-in-all out system, high weaning age, use of hygienic locks, proper disease management, use of hospital pens, and compliance with vaccination protocols have been associated with low infection rates and reduced AMU [18,33,42,49]. Conversely, poor pen conditions, contaminated drinking equipment, poor air quality, and high stocking density have increased AMU [37]. Table 2 (2a and 2b) summarizes the critical farm biosecurity and management factors that have been identified as necessary in reduction (2a) and increase (2b) in AMU across different types of livestock.

Research examining biosecurity measures in calf management has shown that implementing measures such as cleaning and disinfecting calf housing, using dedicated equipment for each calf, ensuring proper colostrum management, implementing vaccination protocols, and monitoring herd health can help decrease AMU by reducing the occurrence of respiratory and gastrointestinal infections in calves [45]. Higher cleaning and disinfection scores, hygienic feed, water, and equipment supply have also been associated with lower resistance to tested antibiotics, suggesting that such biosecurity interventions support AMR mitigation [46]. In addition, high biosecurity farms have been associated with fewer clinical symptoms, lower use of antimicrobials, and better performance [35]. However, animals that move around more frequently and are mixed with animals from different stables without adequate biosecurity measures are more likely to be exposed to germs, leading to an elevated risk of infection [38]. Animal species-specific production issues may also contribute to AMU. For instance, high milk production has been positively correlated with high AMU due to a higher incidence of mastitis [40]. Table 3 outlines the critical farm biosecurity measures and their role in preventing infections and reducing AMU.

In low and middle-income countries (LMICs), raising farmer awareness about the negative effects of untargeted AMU and promoting good farming practices, biosecurity, diagnostic services, and vaccination programs is essential [13]. Studies have shown that proper emphasis on hand hygiene at poultry farms and sensitization about biosecurity management can decrease AMU [43]. Initiatives to better inform farmers and veterinarians on appropriate AMU and farm biosecurity could help reduce AMU on farms [5]. In a study of poultry farms in Belgium, sensitization about biosecurity management with specific advice resulted in a 29% reduction in AMU, as indicated by lower treatment incidences during subsequent audits [32].

The impact of the awareness campaigns (or coaching) and the economic benefits of farm biosecurity interventions in curbing AMU has been assessed in several studies [29,30,31,44]. In a study on Dutch dairy herds, a combination of awareness-raising and restrictive measures was found to reduce antibiotic use by 17% in 2012 compared to 2009 [44]. Another study on raising pigs without antibiotics (RWA) reported that farmers could achieve and maintain RWA status through farm-specific coaching related to prudent AMU and improved biosecurity [29]. A study on pig farms found that implementing new biosecurity measures and vaccinations led to an increase in enterprise profit of +€2.67/finisher pig/year [30]. Also, biosecurity interventions resulted in improved technical results such as the number of weaned piglets/sow/year (+1.1), daily weight gain (+5.9 g/day), and decreased mortality in the finisher period (−0.6%) [31]. These observations provide valuable insights for veterinarians and other stakeholders to encourage livestock farmers to adopt farm biosecurity practices as a cost-effective way to reduce AMU.

It is important to note that not all studies have found a straightforward association between farm biosecurity and reduced AMU. For example, a study on dairy cattle in North-eastern Italy found that there may not be a significant effect of management factors or farm biosecurity on AMU, as the levels of AMU in dairy cattle were not as high as in pig farms in the same region [40]. Similarly, a study on pig farms suggested that there may be a reverse causality effect, where high AMU (due to high disease incidences) may lead to an increase in biosecurity standards, and poor biosecurity may be linked to an increased need for antimicrobial treatments. Furthermore, both AMU and biosecurity can be influenced by various factors (e.g., farm size, animal species, geographic location, etc.), which can act as confounders and mask the association between the two [46]. A study on pig farms revealed a significant link between implementing internal and external biosecurity measures and reducing treatment incidences in pigs. However, when the statistical model analysis included farm and farmer characteristics, this association lost its significance, suggesting the presence of other contributing factors [47]. One possible explanation could be that Swedish herds, which have otherwise good pig health, might have experienced a disease outbreak leading to temporarily high AMU [47]. Moreover, the researchers noted that the tool used to evaluate farm biosecurity may not have been appropriate for Swedish conditions. Thus, when analyzing the findings of studies on-farm biosecurity and AMU, it is vital to consider these subtleties and complexities.

Overall, the literature results suggest that implementing effective farm biosecurity practices and improving farm management practices can reduce AMU on farms or in herds. However, further research is needed to understand these associations’ mechanisms and determine the most effective strategies for promoting and adopting biosecurity practices among farmers.

### 3.3. Interplay between Farm Structure, Management Factors, Biosecurity, and AMU

**Figure 3:**
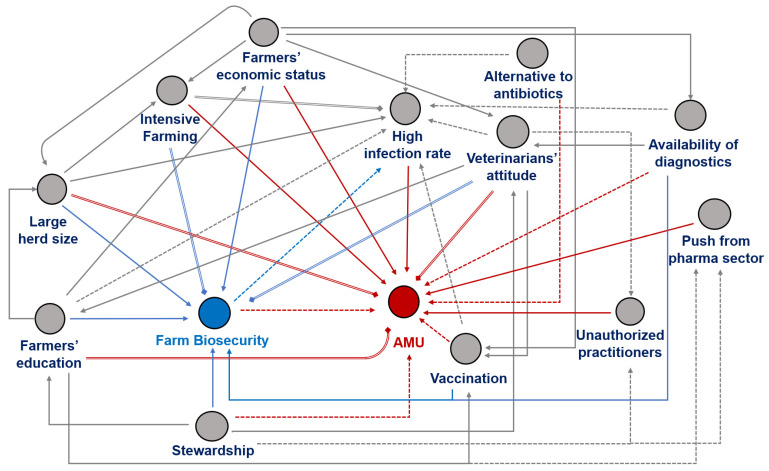
The hypothesized causal diagram illustrates the potential associations between farm biosecurity (or management) factors and antimicrobial usage (AMU). Red lines (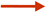 or 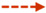 or 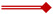) indicate direct association with AMU, blue lines (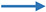 or 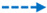 or 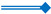) indicate direct association with farm biosecurity, and grey lines (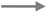 or 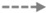 or 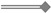) denote possible indirect interactions between factors. A solid arrow (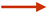 or 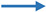 or 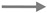) represent increased effects of the factor, a dotted arrow (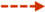 or 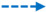 or 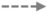) represents decreased effect, and a double-lined rhombus end arrow (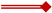 or 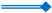 or 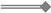) indicates factors with both (increase or decrease) potential effects depending on context.

Through analyzing the literature, it has been established that besides biosecurity, several other farm management factors influence AMU at the herd or farm level. These factors include farm structure, animal health status, disease prevalence or risk of outbreaks, farmers’ socioeconomic and educational status, farmers’ and animal health professionals’ attitudes towards biosecurity and management practices, and regional or national stewardship policies. To illustrate the relationship between these factors and farm biosecurity and AMU, 12 commonly mentioned factors were selected and analyzed. A hypothetical causal diagram was created to show these factors’ direct or indirect effects on farm biosecurity and AMU (Figure 3).

Factors such as biosecurity, vaccination, stewardship, alternatives to antibiotics, and availability of diagnostics can directly affect reducing AMU at the farm level. However, factors such as herd size, farmers’ education, and veterinarians’ attitudes may positively and negatively affect AMU. For example, large herd sizes and livestock species-specific systems can motivate farmers to invest in better management facilities or use antimicrobials for prophylaxis or therapy. Similarly, suppose the farmer is educated more towards infection and treatment perspectives. In that case, there are chances of increased AMU, while education in disease prevention and farm biosecurity would motivate farmers towards decreased use of antimicrobials. The same concept applies to veterinarians’ preferences for therapeutic or preventive aspects.

The relationship between farm size and AMU in livestock production remains complex, and evidence on this topic is mixed. Some studies indicate that among bigger and more intensive farms, biosecurity levels are better as compared to smaller farms, and farmers have more awareness about prudent AMU [9,46]. However, large herds also have several risk factors for disease transmission, and farmers may be more concerned about infections in their herds, leading to increased AMU as prophylaxis or metaphylaxis [50,51,52,53]. Studies in poultry production showed that the increase in herd size could positively impact farm biosecurity practices and a reduction in non-therapeutic usage of antimicrobials, possibly due to contract farming or specific technical inputs from animal health professionals [26,48]. Moreover, herd size may be positively correlated with farmers’ economic condition and educational level, leading to large farms adopting biosecurity measures more efficiently due to better access to resources [33,49,54]. On the other hand, small farms may place less emphasis on management and hygiene procedures, necessitating more treatment to reduce illness rates [55]. Therefore, it is essential to consider other factors, such as management practices and production systems, when assessing the relationship between farm size and AMU. A more refined understanding of this relationship is necessary to develop effective strategies for reducing AMU and promoting sustainable livestock production across all farm sizes.

**Table 3:** Components of farm biosecurity have direct/indirect effects on the infection rate and curbing antimicrobial usage (AMU).

Biosecurity (or Management) Component	Role in Mitigating the Infection Rate and Curbing AMU
**Introduction and movement of livestock**	When adding new animals to the herd, proper “quarantine” methods are essential to preventing the spread of any infections. Also, checking animals’ health status before purchase, segregating suspected animals, and maintaining adequate farm fencing is essential to avoid contact with stray and wild animals. Isomura et al. (2018) observed the effect of ‘better site condition’ and the ‘all-in-all-out’ system on the AMU reduction in pig farms [42]. ‘All-in-all-out’ practice interrupts the routes of disease transmission and thereby reduces the incidence of infections in the herd [56].
**Separation of sick animals**	Separating sick animals helps to stop the infection from spreading, which lowers AMU. A study demonstrated that the farms practising separation of sick birds use significantly less antimicrobials as prophylactic [26].
**Stocking density**	High stocking density is considered a social stressor for the livestock, which might result in decreased performance and an increased risk of infectious diseases. For example, chicken farms with high density and inadequate biosecurity were linked to a higher prevalence of diseases and a rise in AMU [28]. Similarly, researchers have linked organic and extensive pig production systems with low AMU, likely attributed to low animal density and reduced risks associated with confinement [46].
**Colostrum and weaning age**	Adequate quantity and quality of colostrum are crucial for the offspring(s) to fight against infections in the early stages of life and post-natal intestinal development [57]. In addition, researchers have observed that early weaning introduces various stress factors that may influence immune function and intestinal microflora [58]. These disturbances might be associated with the risk of enteric disorders such as post-weaning colibacillosis [59].
**Feed hygiene**	The feed can become contaminated during the production cycle with many pathogens and toxins (e.g., *Salmonella* spp., *Escherichia coli*, *Clostridium* spp., *Aspergillus* spp., and mycotoxins) [33]. Ingestion of contaminated feed may introduce infection in the herd and thereby increase the AMU.
**Transportation**	Improper transportation of animals may cause severe stress (especially young ones). The transportation-associated stressful activities include long-distance associated dehydration and fasting periods, handling of animals during loading and unloading, mixing with unfamiliar groups etc. In addition, animals from different farms brought together might cause stress and increase the risk of infections [60]. Contaminated animal transport vehicles can also spread infectious agents [61]. Therefore, vehicle washing, disinfection, and animal welfare-friendly measures are essential during transportation.
**Farm microclimate**	Poor farm microclimate was associated with a 20% increase in calf mortality, lowering farm profitability by 60% [62,63]. The livestock performance may be significantly impaired by poor air quality parameters, like the accumulation of dust particles, microorganisms and toxins, ammonia, CO_2,_ etc., in the farm environment. Proper ventilation, thorough cleaning of pens, and a reduction in stocking density can help to ensure good air quality in the farms. Researchers found up to 78.9% of AMU was due to respiratory illnesses in young bulls and veal calves [64].
**Accessibility to clean water**	Contaminated water can act as a vehicle for many pathogens, especially associated with enteric disorders. Therefore, water must be stored in a well-closed reservoir to avoid contamination via dust, wild birds, or rodents. Researchers found that farms with shallow water sources for drinking, cleaning, and washing had higher rates of therapeutic use of antimicrobials than deep tube well water [26].
**Cleaning and disinfection**	Routine cleaning and disinfection of farm equipment, waterers, feeders, loading areas, and farm premises can reduce pathogens’ load, vectors (e.g., flies, ticks and mosquitoes) and pollutants that can impair the immune system of livestock [65,66].
**Work routine and separate housing**	Young and newly born animals are more vulnerable to infections than older animals. These infections can be prevented by providing them with separate housing and following working procedures where newborn/young animals are not visited after contact with older animals on the farm [19].
**Carcass, effluent, and waste management**	It is essential to manage the disposal of farm waste and deceased animals appropriately. As the rendering vehicles have previously been linked to the transmission of infections, the cadaver storage room must be situated outside the farm so that the rendering firm can collect the cadavers without accessing the farm [67]. In addition, the environment and public health must be considered when disposing of carcasses and farm waste. The details on various carcass disposal methods are provided in a review by Gwyther et al. (2011) [68].
**Farm personnel and visitor hygiene**	Farm employees and visitors can spread infectious agents to cattle farms by acting as fomites. Due to their frequent interaction with potentially contaminated sources, the boots, clothing, and hands/gloves are at a high risk of becoming fomites. For example, in a study done on poultry farms in the Netherlands, it was observed that the significant transmission pathways of infections for poultry from an external source were staff’s non-adherence to the hygiene standards and not wearing exclusive working clothing before entering the poultry living area [69].

### 3.4. Constraints for Establishing the Association between Biosecurity (or Management) and AMU

Establishing a causal relationship between biosecurity (or management) factors and AMU requires addressing several methodological constraints. For instance, many studies only report the overall effect of a group of biosecurity practices, which makes it difficult to measure the impact of individual interventions [9,38,46]. Moreover, most of the studies are cross-sectional, which limits the ability to infer causality between risk factors and outcomes. Additionally, selection bias is possible due to the voluntary nature of the participation, which may overestimate the effectiveness of interventions [39].

Further, there is limited research on the association between specific biosecurity measures and AMU, particularly in low- and middle-income countries (LMICs) where the burden of animal diseases is high [9]. The use of biosecurity quantification tools is also limited in LMICs, indicating a need for more comprehensive and standardized data collection methods. To establish a robust association between biosecurity (or management) factors and AMU, well-designed intervention field studies controlling for potential confounders and interactions are necessary [32,70].

We acknowledge the limitations of the present study and agree that the specialized nature of farm biosecurity, which involves region, species, and farm-specific factors, makes it challenging to generalize the concept. The exclusion of non-peer-reviewed or grey literature may have resulted in overlooking relevant local or regional content. Additionally, mainly cross-sectional studies were included, limiting the possibility of assessing temporal trends. Moreover, due to insufficient studies, the impact of individual farm biosecurity parameters on AMU could not be conclusively analyzed, although this was not the primary objective of the current review. Nonetheless, we encourage future researchers to consider conducting a meta-analysis when sufficient studies become available in this field.

## 4. Recommendations

Based on the literature analysis, we recommend the following measures to strengthen farm biosecurity and promote judicious AMU.

(a) Review the role of animal health professionals

Effective biosecurity measures require the involvement of all stakeholders in the production chain. Successfully implementing a farm biosecurity protocol or herd health program requires a comprehensive approach that involves setting goals, planning, executing, and evaluating the program. Animal health professionals possess the necessary expertise to oversee and guide these processes, making their role crucial in promoting and implementing farm biosecurity practices to reduce AMU. Therefore, it is recommended to review and enhance the role of animal health professionals in providing support and guidance for farmers to establish effective farm biosecurity protocols and herd health programs [71,72]. Examples of successful collaborations between government, veterinary organizations, and livestock industry stakeholders in the Netherlands resulted in a 56% reduction in AMU in farm animals during 2007–2012 [73]. Therefore, there is a need to foster collaboration between animal health professionals and farmers to develop effective biosecurity plans tailored to individual farms’ specific needs. This may involve providing support and advice to farmers on the selection and use of antimicrobials and other treatments and assisting with implementing biosecurity measures.

(b) Building the farmers’ attitude towards farm biosecurity and judicious AMU

To foster a positive attitude towards farm biosecurity, a change in strategy is necessary as farmers often seek biosecurity-related consulting only when they encounter health problems on their farms and desire quick solutions. The fundamentals of farm biosecurity measures should be presented with regular evaluations by veterinarians and animal health professionals to encourage farmers to appreciate their significance. Additionally, communication of the risks associated with antimicrobial use and resistance is crucial. Conducting a cost-benefit analysis to demonstrate the overall benefits of biosecurity interventions over prophylactic use of antimicrobials is also recommended. To overcome the barriers to adopting biosecurity practices, it may be necessary to provide financial incentives or support and tailored information and resources.

(c) Advocating the adoption of biosecurity quantification tools

Encouraging farmers and veterinarians to use biosecurity quantification tools can aid in identifying and prioritizing biosecurity risks on farms. Training and support for these tools can promote their adoption, and continued research and development can ensure their effectiveness in addressing biosecurity risks associated with antimicrobial use. Collaborative efforts between farmers, veterinarians, and researchers can help to ensure that these tools are relevant and practical for use in different farming systems and contexts.

(d) Development of monitoring and surveillance system for AMU

The availability of quantitative data on AMU in European countries is due to their well-established monitoring and surveillance systems. The details on these systems can be accessed through the AACTING network, which stands for ‘Network on quantification of veterinary Antimicrobial usage at herd level and Analysis, CommunicaTion and benchmarkING to improve responsible usage’ “weblink: https://aacting.org/about-aacting/ (accessed on 8 March 2023)”. However, most low- and middle-income countries (LMICs) lack centralized recording of AMU in food animals, and data sources are limited to aggregated data of imports or sales. This leads to a lack of detailed information on quantities used in various animal species or locations. Despite minimal regulation of antimicrobials in small-scale production systems in LMICs, research on AMU in these systems remains limited. Further, to effectively monitor and compare AMU, standardized definitions and calculation methods are necessary, and data transfer should be streamlined. Therefore, there is a need for increased efforts to fill these gaps and improve data availability and comparability across countries.

## 5. Conclusions

The review has provided valuable insights into the link between farm biosecurity and AMU, and it confirms that biosecurity plays a vital role in the effort to reduce AMU. The study findings suggest a lack of emphasis on quantifying farm biosecurity and herd health management practices. Most studies conducted in European regions focused on piggeries and poultry farms, indicating a knowledge gap in LMICs. Most studies were cross-sectional, highlighting the need for longitudinal studies to establish strong evidence on the targeted associations. The limited application of region- and species-specific quantitative biosecurity scoring systems and AMU monitoring systems outside of Europe suggest applying tools for categorising farm biosecurity and benchmarking antimicrobial consumption to formulate an evidence-based sustainable plan for judicious AMU. Further research is necessary to understand the interrelatedness of biosecurity parameters and their impact on herd health, farm production, and AMU. LMIC stakeholders and policymakers need to be made aware of the benefits of adopting farm biosecurity, and field-level studies can be conducted to establish this. Finally, additional research is required to develop evidence-based guidelines for farmers to promote optimal farm biosecurity practices and antimicrobial usage.

## Figures and Tables

**Table 2:** Farm biosecurity and management factors were observed in this review to be associated with AMU among various livestock species.

**2a: Biosecurity or Management Factors Related to a Reduction in Antimicrobial Usage (AMU)**		
**Species**	**Internal (Biosecurity or Management) Factors**	**External (Biosecurity or Management) Factors**
Pigs	▪ All-in and all-out system at all stages ▪ Complete control of pig flow ▪ Farms with low stocking density ▪ Disinfection of the loading area ▪ Weaning age > 24 days ▪ High cleaning and disinfection score ▪ Control of feed, water and equipment supply ▪ Pen cleanliness ▪ Well-ventilated farm ▪ Work line from younger pigs to older ones ▪ Hygienic drinking equipment ▪ Anthelmintic therapy ▪ Vaccination ▪ Disease management (i.e., use of hospital pens, handling of diseased animals) ▪ Rapid diagnostic methods	▪ Low chance of having other herds located within a radius of 500 m ▪ Proper quarantine measures for new animals brought onto the farm ▪ Organic-extensive farms ▪ Pest control
Poultry	▪ Separation of sick from healthy birds ▪ Stable-specific clothing ▪ House-specific and recognizable materials and farm clothing ▪ Cleaning the drinking water system after every production round ▪ Having proper vaccination protocols ▪ Use of personal protective equipment ▪ Low chicken morbidity ▪ Vermin control program ▪ Disinfection of stables after every production round	▪ Farmers with large flocks and high experience and education ▪ Footbath at the entrance ▪ Farm hygiene lock ▪ Hygiene protocol before and after entering the farm ▪ Hand washing before and after entering the farm ▪ Use of exclusive working clothes by staff and visitors ▪ Proper mortality disposal strategy
Cattle	▪ Age-specific management ▪ Cleaning and disinfection of barn ▪ Use of replaceable bedding materials (e.g., straw, sawdust etc.) ▪ Proper udder health management	▪ Avoidance of contact with other herds, such as fencing or avoiding shared grazing areas ▪ Quarantine measures for new animals brought onto the farm
**2b: Biosecurity or Management Factors Related to the Increase in Antimicrobial Usage (AMU)**		
**Species**	**Internal (Biosecurity or Management) Factors**	**External (Biosecurity or Management) Factors**
Pigs	▪ Intensive farms with large herds ▪ Specialized fattening farms ▪ Shorter farrowing rhythm ▪ Younger weaning age ▪ Poor air quality causes respiratory diseases ▪ Post-weaning mortality risk, lameness, tail biting, gastrointestinal diseases, and skin symptoms in the herd ▪ Pneumonia and oedema disease in piglets	▪ Increased animal movement and pooling of animals from different stables
Poultry	▪ The high number of broilers per round ▪ Flock group treatments among broilers ▪ High chicken morbidity ▪ Performing flock depopulation in two or more steps	▪ The high number of farm workers and visitors ▪ Farms using shallow water as compared to the deep tube well
Cattle	▪ Low hygiene during milking routines ▪ Housing calves on partially slatted floors ▪ Negligence of udder health ▪ Respiratory and gastrointestinal problems in calves	▪ Presence of vectors and pests in the region ▪ Advocacy for high AMU to address the prevention of infections during the dry period and treatment of subclinical and clinical mastitis

## Supplementary Materials

The following supporting information can be downloaded at: https://www.mdpi.com/article/10.3390/antibiotics12050893/s1, Supplementary Table S1: Preferred Reporting Items for Systematic Reviews and Meta-Analyses extension for Scoping Reviews (PRISMA-ScR) Checklist. Supplementary Table S2: Search approaches for scoping review for the research question “Is There an Association Between Antimicrobial Usage and Farm Biosecurity in Food Animals?”: search strategy applied in PubMed.

## References

[1] Cook M.A., Wright G.D. (2022). The past, present, and future of antibiotics. Sci. Transl. Med..

[2] Friedman M., Friedland G.W. (1998). Medicine’s 10 Greatest Discoveries.

[3] Low C.X., Tan L.T.-H., Ab Mutalib N.-S., Pusparajah P., Goh B.-H., Chan K.-G., Letchumanan V., Lee L.-H. (2021). Unveiling the Impact of Antibiotics and Alternative Methods for Animal Husbandry: A Review. Antibiotics.

[4] Ikhimiukor O.O., Odih E.E., Donado-Godoy P., Okeke I.N. (2022). A bottom-up view of antimicrobial resistance transmission in developing countries. Nat. Microbiol..

[5] Pires J., Huber L., Hickman R.A., Dellicour S., Lunha K., Leangapichart T., Jiwakanon J., Magnusson U., Sunde M., Järhult J.D. (2022). Genome-associations of extended-spectrum ß-lactamase producing (ESBL) or AmpC producing E. coli in small and medium pig farms from Khon Kaen province, Thailand. BMC Microbiol..

[6] Tang K.L., Caffrey N.P., Nóbrega D.B., Cork S.C., Ronksley P.E., Barkema H.W., Polachek A.J., Ganshorn H., Sharma N., Kellner J.D. (2017). Restricting the use of antibiotics in food-producing animals and its associations with antibiotic resistance in food-producing animals and human beings: A systematic review and meta-analysis. Lancet Planet. Health.

[7] Zhang Q.-Q., Ying G.-G., Pan C.-G., Liu Y.-S., Zhao J.-L. (2015). Comprehensive Evaluation of Antibiotics Emission and Fate in the River Basins of China: Source Analysis, Multimedia Modeling, and Linkage to Bacterial Resistance. Environ. Sci. Technol..

[8] Ma F., Xu S., Tang Z., Li Z., Zhang L. (2021). Use of antimicrobials in food animals and impact of transmission of antimicrobial resistance on humans. Biosaf. Health.

[9] Mallioris P., Teunis G., Lagerweij G., Joosten P., Dewulf J., Wagenaar J.A., Stegeman A., Mughini-Gras L. (2022). Biosecurity, and antimicrobial use in broiler farms across nine European countries: Towards identifying farm-specific options for reducing antimicrobial usage. Epidemiol. Infect..

[10] Ungemach F.R., Müller-Bahrdt D., Abraham G. (2006). Guidelines for prudent use of antimicrobials and their implications on antibiotic usage in veterinary medicine. Int. J. Med. Microbiol..

[11] Holmes A.H., Moore L.S.P., Sundsfjord A., Steinbakk M., Regmi S., Karkey A., Guerin P.J., Piddock L.J.V. (2016). Understanding the mechanisms and drivers of antimicrobial resistance. Lancet.

[12] Lhermie G., Gröhn Y.T., Raboisson D. (2017). Addressing Antimicrobial Resistance: An Overview of Priority Actions to Prevent Suboptimal Antimicrobial Use in Food-Animal Production. Front. Microbiol..

[13] Luu Q.H., Nguyen T.L.A., Pham T.N., Vo N.G., Padungtod P. (2021). Antimicrobial use in household, semi-industrialized, and industrialized pig and poultry farms in Viet Nam. Prev. Vet. Med..

[14] Dutra M.C., Moreno L.Z., Dias R.A., Moreno A.M. (2021). Antimicrobial Use in Brazilian Swine Herds: Assessment of Use and Reduction Examples. Microorganisms.

[15] Teillant A., Brower C.H., Laxminarayan R. (2015). Economics of Antibiotic Growth Promoters in Livestock. Annu. Rev. Resour. Econ..

[16] Joosten P., Sarrazin S., Van Gompel L., Luiken R.E.C., Mevius D.J., Wagenaar J.A., Heederik D.J.J., Dewulf J. (2019). EFFORT consortium Quantitative and qualitative analysis of antimicrobial usage at farm and flock level on 181 broiler farms in nine European countries. J. Antimicrob. Chemother..

[17] Sarrazin S., Joosten P., Van Gompel L., Luiken R.E.C., Mevius D.J., Wagenaar J.A., Heederik D.J.J., Dewulf J. (2019). Quantitative and qualitative analysis of antimicrobial usage patterns in 180 selected farrow-to-finish pig farms from nine European countries based on single batch and purchase data. J. Antimicrob. Chemother..

[18] Postma M., Backhans A., Collineau L., Loesken S., Sjölund M., Belloc C., Emanuelson U., Grosse Beilage E., Nielsen E.O., Stärk K.D.C. (2016). Evaluation of the relationship between the biosecurity status, production parameters, herd characteristics and antimicrobial usage in farrow-to-finish pig production in four EU countries. Porc. Health Manag..

[19] Dewulf J., Van Immerseel F. (2019). Biosecurity in Animal Production and Veterinary Medicine.

[20] Robertson I.D. (2020). Disease Control, Prevention and On-Farm Biosecurity: The Role of Veterinary Epidemiology. Engineering.

[21] Pritchard G., Dennis I., Waddilove J. (2005). Biosecurity: Reducing disease risks to pig breeding herds. Practice.

[22] Caekebeke N., Jonquiere F.J., Ringenier M., Tobias T.J., Postma M., van den Hoogen A., Houben M.A.M., Velkers F.C., Sleeckx N., Stegeman J.A. (2020). Comparing Farm Biosecurity and Antimicrobial Use in High-Antimicrobial-Consuming Broiler and Pig Farms in the Belgian–Dutch Border Region. Front. Vet. Sci..

[23] Albernaz-Gonçalves R., Olmos G., Hötzel M.J. (2021). Exploring Farmers’ Reasons for Antibiotic Use and Misuse in Pig Farms in Brazil. Antibiotics.

[24] Higgins H.M., Mouncey J., Nanjiani I., Cook A.J.C. (2017). Understanding how new evidence influences practitioners’ beliefs regarding dry cow therapy: A Bayesian approach using probabilistic elicitation. Prev. Vet. Med..

[25] Maye D., Chan K.W. (2020). (Ray) On-farm biosecurity in livestock production: Farmer behaviour, cultural identities, and practices of care. Emerg. Top. Life Sci..

[26] Imam T., Gibson J.S., Gupta S.D., Hoque M.A., Fournié G., Henning J. (2021). Association between farm biosecurity practices and antimicrobial usage on commercial chicken farms in Chattogram, Bangladesh. Prev. Vet. Med..

[27] Tricco A.C., Lillie E., Zarin W., O’Brien K.K., Colquhoun H., Levac D., Moher D., Peters M.D.J., Horsley T., Weeks L. (2018). PRISMA Extension for Scoping Reviews (PRISMA-ScR): Checklist and Explanation. Ann. Intern. Med..

[28] Chowdhury S., Fournié G., Blake D., Henning J., Conway P., Hoque M.A., Ghosh S., Parveen S., Biswas P.K., Akhtar Z. (2022). Antibiotic usage practices and its drivers in commercial chicken production in Bangladesh. PLoS ONE.

[29] Bernaerdt E., Maes D., Van Limbergen T., Postma M., Dewulf J. (2022). Determining the Characteristics of Farms That Raise Pigs without Antibiotics. Animals.

[30] Rojo-Gimeno C., Postma M., Dewulf J., Hogeveen H., Lauwers L., Wauters E. (2016). Farm-economic analysis of reducing antimicrobial use whilst adopting improved management strategies on farrow-to-finish pig farms. Prev. Vet. Med..

[31] Postma M., Vanderhaeghen W., Sarrazin S., Maes D., Dewulf J. (2017). Reducing Antimicrobial Usage in Pig Production without Jeopardizing Production Parameters. Zoonoses Public Health.

[32] Gelaude P., Schlepers M., Verlinden M., Laanen M., Dewulf J. (2014). Biocheck.UGent: A quantitative tool to measure biosecurity at broiler farms and the relationship with technical performances and antimicrobial use. Poult. Sci..

[33] Laanen M., Persoons D., Ribbens S., de Jong E., Callens B., Strubbe M., Maes D., Dewulf J. (2013). Relationship between biosecurity and production/antimicrobial treatment characteristics in pig herds. Vet. J. Lond. Engl. 1997.

[34] Horie M., Yang D., Joosten P., Munk P., Wadepohl K., Chauvin C., Moyano G., Skarżyńska M., Dewulf J., Aarestrup F.M. (2021). Risk Factors for Antimicrobial Resistance in Turkey Farms: A Cross-Sectional Study in Three European Countries. Antibiot. Basel Switz..

[35] Collineau L., Backhans A., Dewulf J., Emanuelson U., Grosse Beilage E., Lehébel A., Loesken S., Okholm Nielsen E., Postma M., Sjölund M. (2017). Profile of pig farms combining high performance and low antimicrobial usage within four European countries. Vet. Rec..

[36] Luiken R.E.C., Van Gompel L., Munk P., Sarrazin S., Joosten P., Dorado-García A., Borup Hansen R., Knudsen B.E., Bossers A., Wagenaar J.A. (2019). Associations between antimicrobial use and the faecal resistome on broiler farms from nine European countries. J. Antimicrob. Chemother..

[37] Stygar A.H., Chantziaras I., Toppari I., Maes D., Niemi J.K. (2020). High biosecurity and welfare standards in fattening pig farms are associated with reduced antimicrobial use. Anim. Int. J. Anim. Biosci..

[38] Hemme M., Ruddat I., Hartmann M., Werner N., van Rennings L., Käsbohrer A., Kreienbrock L. (2018). Antibiotic use on German pig farms—A longitudinal analysis for 2011, 2013 and 2014. PLoS ONE.

[39] Raasch S., Postma M., Dewulf J., Stärk K.D.C., Grosse Beilage E. (2018). Association between antimicrobial usage, biosecurity measures as well as farm performance in German farrow-to-finish farms. Porc. Health Manag..

[40] Menegon F., Capello K., Tarakdjian J., Pasqualin D., Cunial G., Andreatta S., Dellamaria D., Manca G., Farina G., Di Martino G. (2022). Antibiotic Use in Alpine Dairy Farms and Its Relation to Biosecurity and Animal Welfare. Antibiot. Basel Switz..

[41] Scoppetta F., Sensi M., Franciosini M.P., Capuccella M. (2017). Evaluation of antibiotic usage in swine reproduction farms in Umbria region based on the quantitative analysis of antimicrobial consumption. Ital. J. Food Saf..

[42] Isomura R., Matsuda M., Sugiura K. (2018). An epidemiological analysis of the level of biosecurity and animal welfare on pig farms in Japan and their effect on the use of veterinary antimicrobials. J. Vet. Med. Sci..

[43] Ornelas-Eusebio E., García-Espinosa G., Laroucau K., Zanella G. (2020). Characterization of commercial poultry farms in Mexico: Towards a better understanding of biosecurity practices and antibiotic usage patterns. PLoS ONE.

[44] Kuipers A., Koops W.J., Wemmenhove H. (2016). Antibiotic use in dairy herds in the Netherlands from 2005 to 2012. J. Dairy Sci..

[45] Holstege M.M.C., de Bont-Smolenaars A.J.G., Santman-Berends I.M.G.A., van der Linde-Witteveen G.M., van Schaik G., Velthuis A.G.J., Lam T.J.G.M. (2018). Factors associated with high antimicrobial use in young calves on Dutch dairy farms: A case-control study. J. Dairy Sci..

[46] Mencía-Ares O., Argüello H., Puente H., Gómez-García M., Manzanilla E.G., Álvarez-Ordóñez A., Carvajal A., Rubio P. (2021). Antimicrobial resistance in commensal Escherichia coli and Enterococcus spp. is influenced by production system, antimicrobial use, and biosecurity measures on Spanish pig farms. Porc. Health Manag..

[47] Backhans A., Sjölund M., Lindberg A., Emanuelson U. (2016). Antimicrobial use in Swedish farrow-to-finish pig herds is related to farmer characteristics. Porc. Health Manag..

[48] Bâtie C., Ha L.T.T., Loire E., Truong D.B., Tuan H.M., Cuc N.T.K., Paul M., Goutard F. (2022). Characterisation of chicken farms in Vietnam: A typology of antimicrobial use among different production systems. Prev. Vet. Med..

[49] Mallioris P., Dohmen W., Luiken R.E.C., Wagenaar J.A., Stegeman A., Mughini-Gras L. (2022). Factors associated with antimicrobial use in pig and veal calf farms in the Netherlands: A multi-method longitudinal data analysis. Prev. Vet. Med..

[50] Bos M.E.H., Taverne F.J., van Geijlswijk I.M., Mouton J.W., Mevius D.J., Heederik D.J.J., on behalf of the Netherlands Veterinary Medicines Authority (SDa) (2013). Consumption of Antimicrobials in Pigs, Veal Calves, and Broilers in The Netherlands: Quantitative Results of Nationwide Collection of Data in 2011. PLoS ONE.

[51] Gardner I.A., Willeberg P., Mousing J. (2002). Empirical and theoretical evidence for herd size as a risk factor for swine diseases. Anim. Health Res. Rev..

[52] Lekagul A., Tangcharoensathien V., Yeung S. (2018). The use of antimicrobials in global pig production: A systematic review of methods for quantification. Prev. Vet. Med..

[53] Woolums A.R., Berghaus R.D., Smith D.R., White B.J., Engelken T.J., Irsik M.B., Matlick D.K., Jones A.L., Ellis R.W., Smith I.J. (2013). Producer survey of herd-level risk factors for nursing beef calf respiratory disease. J. Am. Vet. Med. Assoc..

[54] Bokma J., Dewulf J., Deprez P., Pardon B. (2018). Risk factors for antimicrobial use in food-producing animals: Disease prevention and socio-economic factors as the main drivers?. Vlaams Diergeneeskd. Tijdschr..

[55] Vieira A.R., Pires S.M., Houe H., Emborg H.-D. (2011). Trends in slaughter pig production and antimicrobial consumption in Danish slaughter pig herds, 2002–2008. Epidemiol. Infect..

[56] Eriksen E.Ø., Pedersen K.S., Larsen I., Nielsen J.P. (2022). Evidence-Based Recommendations for Herd Health Management of Porcine Post-Weaning Diarrhea. Animals.

[57] Hammon H.M., Liermann W., Frieten D., Koch C. (2020). Review: Importance of colostrum supply and milk feeding intensity on gastrointestinal and systemic development in calves. Animal.

[58] Blavi L., Solà-Oriol D., Llonch P., López-Vergé S., Martín-Orúe S.M., Pérez J.F. (2021). Management and Feeding Strategies in Early Life to Increase Piglet Performance and Welfare around Weaning: A Review. Animals.

[59] Wellock I.J., Fortomaris P.D., Houdijk J.G.M., Kyriazakis I. (2008). Effects of dietary protein supply, weaning age and experimental enterotoxigenic Escherichia coli infection on newly weaned pigs: Performance. Anim. Int. J. Anim. Biosci..

[60] Pardon B., Catry B., Dewulf J., Persoons D., Hostens M., De Bleecker K., Deprez P. (2012). Prospective study on quantitative and qualitative antimicrobial and anti-inflammatory drug use in white veal calves. J. Antimicrob. Chemother..

[61] Lowe J., Gauger P., Harmon K., Zhang J., Connor J., Yeske P., Loula T., Levis I., Dufresne L., Main R. (2014). Role of Transportation in Spread of Porcine Epidemic Diarrhea Virus Infection, United States. Emerg. Infect. Dis..

[62] Gauly M., Bollwein H., Breves G., Brügemann K., Dänicke S., Daş G., Demeler J., Hansen H., Isselstein J., König S. (2013). Future consequences and challenges for dairy cow production systems arising from climate change in Central Europe—A review. Animal.

[63] Herbut P. (2010). Air movement characteristics inside a cow barn with natural ventilation under no-wind conditions in the winter season. Infrastruktura Ekol. Teren. Wiej..

[64] Fertner M., Toft N., Martin H.L., Boklund A. (2016). A register-based study of the antimicrobial usage in Danish veal calves and young bulls. Prev. Vet. Med..

[65] De Busser E.V., De Zutter L., Dewulf J., Houf K., Maes D. (2013). Salmonella control in live pigs and at slaughter. Vet. J..

[66] Rasschaert G., De Zutter L., Herman L., Heyndrickx M. (2020). Campylobacter contamination of broilers: The role of transport and slaughterhouse. Int. J. Food Microbiol..

[67] McQuiston J.H., Garber L.P., Porter-Spalding B.A., Hahn J.W., Pierson F.W., Wainwright S.H., Senne D.A., Brignole T.J., Akey B.L., Holt T.J. (2005). Evaluation of risk factors for the spread of low pathogenicity H7N2 avian influenza virus among commercial poultry farms. J. Am. Vet. Med. Assoc..

[68] Gwyther C.L., Williams A.P., Golyshin P.N., Edwards-Jones G., Jones D.L. (2011). The environmental and biosecurity characteristics of livestock carcass disposal methods: A review. Waste Manag..

[69] Ssematimba A., Hagenaars T.J., de Wit J.J., Ruiterkamp F., Fabri T.H., Stegeman J.A., de Jong M.C.M. (2013). Avian influenza transmission risks: Analysis of biosecurity measures and contact structure in Dutch poultry farming. Prev. Vet. Med..

[70] Rodrigues da Costa M., Gasa J., Calderón Díaz J.A., Postma M., Dewulf J., McCutcheon G., Manzanilla E.G. (2019). Using the Biocheck.UGent^TM^ scoring tool in Irish farrow-to-finish pig farms: Assessing biosecurity and its relation to productive performance. Porc. Health Manag..

[71] Derks M., van de Ven L.M.A., van Werven T., Kremer W.D.J., Hogeveen H. (2012). The perception of veterinary herd health management by Dutch dairy farmers and its current status in the Netherlands: A survey. Prev. Vet. Med..

[72] Jansen J., Steuten C.D.M., Renes R.J., Aarts N., Lam T.J.G.M. (2010). Debunking the myth of the hard-to-reach farmer: Effective communication on udder health. J. Dairy Sci..

[73] Speksnijder D.C., Mevius D.J., Bruschke C.J.M., Wagenaar J.A. (2015). Reduction of veterinary antimicrobial use in the Netherlands. The Dutch success models. Zoonoses Public Health.

