# Supplementary material for: Can Improved Farm Biosecurity Reduce the Need for Antimicrobials in Food Animals? A Scoping Review
Source: Antibiotics (Basel). 2023 May 11;12(5):893. doi: 10.3390/antibiotics12050893 (PMC10215210; doi:10.3390/antibiotics12050893)
Supplement: Supplementary file 1 [file antibiotics-12-00893-s001.zip › Supplementary Table S2_Search strategy.pdf]

**Supplementary Table S2: Search approaches for scoping review for the research question "Is There an Association Between Antimicrobial Usage and Farm Biosecurity in Food Animals?": search strategy applied in PubMed**

| | |
|---|---|
| *Population* | |
| 1 | "Livestock" |
| 2 | "poultry" |
| 3 | "cattle" |
| 4 | "dairy animals" |
| 5 | "dairy cattle" |
| 6 | "beef production" |
| 7 | "beef cattle" |
| 8 | "buffaloes" |
| 9 | "pig" |
| 10 | "swine" |
| 11 | "goat" |
| 12 | "sheep" |
| 13 | "chicken" |
| 14 | "broiler" |
| 15 | "layer" |
| 16 | "turkey" |
| 17 | "duck" |
| 18 | ("Livestock" OR "poultry" OR "farm" OR "animal production" OR "cattle" OR "dairy animals" OR "dairy cattle" OR "beef production" OR "beef cattle" OR "buffalo*" OR "pig" OR "swine" OR "goat" OR "sheep" OR "chicken" OR "broiler" OR "layer" OR "turkey" OR "duck") |
| 19 | OR/1-17 |
| *Area of Interest* | |
| 20 | "antimicrobial resistance" |
| 21 | "antibiotic resistance" |
| 22 | "antimicrobial usage" |
| 23 | "antibiotic usage" |
| 24 | ("antimicrobial resistance" OR "antibiotic resistance" OR "antimicrobial usage" OR "antibiotic usage") |
| 25 | OR/20-23 |
| *Context* | |
| 26 | "biosecurity" |
| 27 | "farm biosecurity" |
| 28 | "animal biosecurity" |
| 29 | "preventive veterinary medicine" |
| 30 | "herd health management" |
| 31 | ("biosecurity" OR "farm biosecurity" OR "animal biosecurity" OR "preventive veterinary medicine" OR "herd health management") |
| 32 | OR/26-30 |
| *Outcomes* | |

| | |
|---|---|
| 33 | ("biosecurity” OR farm biosecurity" OR "animal biosecurity" OR "preventive veterinary medicine" OR "herd health management") |
| 34 | ("antimicrobial resistance" OR "antibiotic resistance" OR "antimicrobial usage" OR "antibiotic usage") |
| 35 | ("Livestock" OR "poultry" OR "farm" OR "animal production" OR "cattle" OR "dairy animals" OR "dairy cattle" OR "beef production" OR "beef cattle" OR "buffalo*" OR "pig" OR "swine" OR "goat" OR "sheep" OR "chicken" OR "broiler" OR "layer" OR "turkey" OR "duck") |
| 36 | (“Biosecurity” OR "farm biosecurity" OR "animal biosecurity" OR "preventive veterinary medicine" OR "herd health management") AND ("antimicrobial resistance" OR "antibiotic resistance" OR "antimicrobial usage" OR "antibiotic usage") AND ("Livestock" OR "poultry" OR "farm" OR "animal production" OR "cattle" OR "dairy animals" OR "dairy cattle" OR "beef production" OR "beef cattle" OR "buffalo*" OR "pig" OR "swine" OR "goat" OR "sheep" OR "chicken" OR "broiler" OR "layer" OR "turkey" OR "duck") |
| 37 | AND/18, 24, 31 |
